# Intracameral air injection during Ahmed glaucoma valve implantation in neovascular glaucoma for the prevention of tube obstruction with blood clot: Case Report

**DOI:** 10.1097/MD.0000000000009092

**Published:** 2017-12-15

**Authors:** Sung Ha Hwang, Chungkwon Yoo, Yong Yeon Kim, Dae Young Lee, Dong Heun Nam, Jong Yeon Lee

**Affiliations:** a Department of Ophthalmology, Gachon University, College of Medicine Gil Medical Center, Incheon; b Department of Ophthalmology, Korea University College of Medicine, Seoul, Republic of Korea.

**Keywords:** Ahmed glaucoma valve implantation, air, intraocular pressure, neovascular glaucoma, tube obstruction with blood clot

## Abstract

**Rationale:**

Glaucoma drainage implant surgery is a treatment option for the management of neovascular glaucoma. However, tube obstruction by blood clot after Ahmed glaucoma valve (AGV) implantation is an unpredictable clinically challenging situation.

**Patient concerns-diagnoses-interventions:**

We report 4 cases using intracameral air injection for the prevention of the tube obstruction of AGV by blood clot.

**Outcomes:**

The first case was a 57-year-old female suffering from ocular pain because of a tube obstruction with blood clot after AGV implantation in neovascular glaucoma. Surgical blood clot removal was performed. However, intractable bleeding was noted during the removal of the blood clot, and so intracameral air injection was performed to prevent a recurrent tube obstruction. After the procedure, although blood clots formed around the tube, the tube opening where air could touch remained patent. In 3 cases of neovascular glaucoma with preoperative severe intraocular hemorrhages, intracameral air injection and AGV implantation were performed simultaneously. In all 3 cases, tube openings were patent. It appears that air impeded the blood clots formation in front of the tube opening.

**Lessons:**

Intracameral air injection could be a feasible option to prevent tube obstruction of AGV implant with a blood clot in neovascular glaucoma with high risk of tube obstruction.

## 1 Introduction

Glaucoma drainage implant surgery is a treatment option for the management of refractory glaucoma, such as neovascular glaucoma. However, during glaucoma drainage implant surgery, hyphema can develop due to a sudden change in intraocular pressure (IOP) or damage of neovascularization of anterior chamber angle during sclerotomy or tube insertion, especially in the presence of neovascular glaucoma or inflammatory glaucoma. Serious hyphema that obstructs the tube lumen results in sustained IOP elevation postoperatively in eyes with an already compromised optic nerve. The tube obstruction in the postoperative course has been reported with incidence of 5% to 11% in previous studies.^[1–6]^ Antivascular endothelial growth factor (anti-VEGF) injection may reduce postoperative bleeding risk.^[7–10]^ However, the tube obstruction rate was reported to be not reduced.^[11]^ Therefore, complete tube obstruction by blood clots after the surgery is still an unpredictable challenge to glaucoma specialists.

Current managements for tube obstruction by blood clots include steroid use, tissue plasminogen activator (T-PA), or surgical removal.^[12]^ However, topical and oral steroid may need time to resolve clots and may fail in some cases. T-PA may be effective, but complications of massive hyphema, flat anterior chamber, and hypotony have been reported.^[12]^ Furthermore, surgical removal of blood clot still take a risk of recurrent tube obstruction by bleeding. Thus, a more effective method is required to prevent tube obstruction by a blood clot, especially in high risk cases. Therefore, we describe cases using a feasible surgical technique for the prevention of tube obstruction of Ahmed glaucoma valve (AGV) implant (New World Medical, Rancho Cucamonga, CA) by blood clots where intracameral air injection was performed. One case of postoperative tube obstruction by a blood clot after AGV implant placement was managed by clot removal and intracameral air injection to prevent the recurrent tube obstruction. The other 3 neovascular glaucoma cases had serious preoperative intraocular hemorrhages, and in these cases, intracameral air injection was performed simultaneously with AGV implantation to prevent tube obstruction with blood clots.

## 2 Methods

A retrospective chart review was performed on 4 patients that underwent intracameral air injection and glaucoma shunt surgery to prevent tube obstruction by blood clots in neovascular glaucoma and hyphema. We used the same AGV implant (model FP7) for all cases. At the end of AGV implantation, filtered air was injected into the anterior chamber through the paracentesis with a 30 gauge needle to keep the tube of the AGV implant in an air bubble. IOP of each case at every time point was measured by Goldmann applanation tonometry. This study was approved by the institutional review board and followed the ethical standards of the Declaration of Helsinki.

### 2.1 Case 1

**Figure 1:**
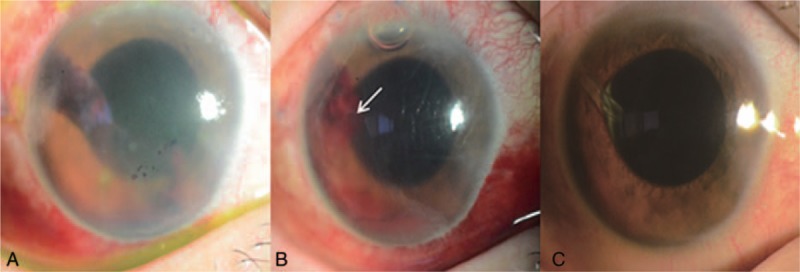
Case 1: 57/F, (A) Tube obstruction by blood clots on the first day after Ahmed glaucoma valve (AGV) implantation for neovascular glaucoma. (B) On the first day after clot removal with intracameral air injection, although blood clots were observed in the peritubal area, the tube opening remained patent (white arrow). (C) Blood clots around the tube and hyphema were disappeared with stable IOP in 2 weeks after clot removal with intracameral air injection. AGV = Ahmed glaucoma valve.

A 57-year-old woman was referred to our clinic for ocular pain and visual disturbance of the right eye. The patient had an ophthalmologic history of vitrectomy and cataract surgery of the right eye for central retinal vein occlusion, a macular hole, and cataract. She also has diabetes and hypertension. Her last visit was 3 years before this referral, and she had been lost to a regular follow-up. Best corrected visual acuity (BCVA) of the right eye was hand motion and IOP was 42 mm Hg. A diagnosis of neovascular glaucoma of the right eye was based on extensive peripheral anterior synechiae (PAS) and neovascularization of iris (NVI). Intravitreal anti-VEGF injection and maximal tolerated medical therapy (MTMT) were ineffective, and thus, AGV implantation was performed. IOP on the first postoperative day was 33 mm Hg and visual acuity was light perception. In addition, severe blood clot formation was observed on the peritubal area of the AGV (Fig. 1A). Since clots blocked the tube lumen, they were removed by tube irrigation with BSS. However, intractable hyphema was observed in anterior chamber and some peritubal blood clots were not completely removed because they were firmly attached to the outer surface of the tube. Due to the risk of recurrent tube obstruction, we injected air into the anterior chamber with about 50% air fill assuming that the air in front of the tube opening would prevent blood clots formation in the tube opening. The patient was instructed to adopt a head up position. The day after the procedure, intraocular pressure was 15 mm Hg and remnant air was observed in the superior part of anterior chamber. Although a blood clot was observed in the peritubal area, the tube opening remained patent (Fig. 1B). On the 2 weeks after intracameral air injection and tube irrigation, intraocular pressure was 15 mm Hg and visual acuity had increased to 0.06 (Snellen), and no blood clot was evident in the anterior chamber (Fig. 1C). During the 6 months after the surgery, her intraocular pressure remained stable under 3 topical medications without any other complications (Tables 1 and 2).

**Table 1:**
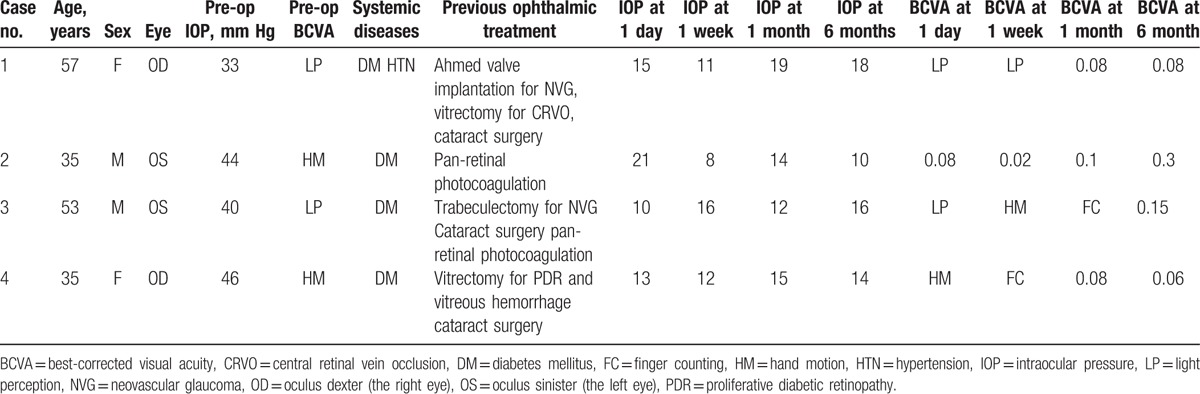
Intraocular pressure and visual acuity in each case over the follow-up period.

**Table 2:**
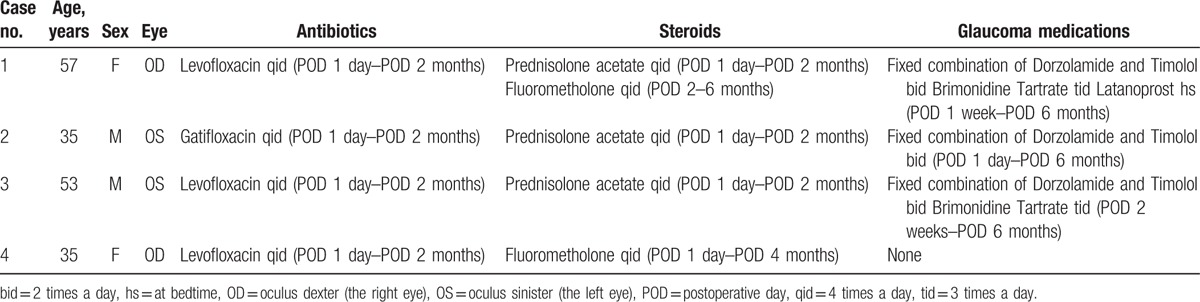
Postoperative topical medications in each case over the follow-up period.

### 2.2 Case 2

**Figure 2:**
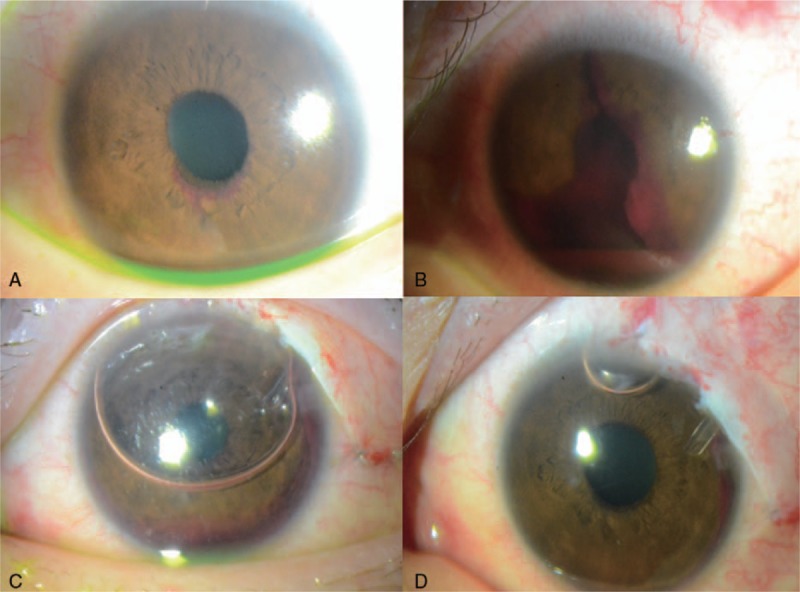
Case 2: 35/M, (A) neovascular glaucoma was diagnosed with high IOP and iris of neovascularization. (B) Severe hyphema was developed on the first day after anti-VEGF injection. (C) On the first day after AGV implantation and intracameral air injection, air pushed blood away from the tube and blood clot was formed on the periphery of anterior chamber. (D) At 3 days after the surgery, air decreased and IOP was normalized. AGV = Ahmed glaucoma valve, IOP = intraocular pressure, VEGF = vascular endothelial growth factor.

A 35-year-old man was referred with the complaint of ocular pain of the left eye. He had a history of pan-retinal photocoagulation and intravitreal anti-VEGF injection due to proliferative diabetic retinopathy and macular edema in both eyes. Right eye BCVA was 0.6 and left eye finger count at 50 cm. IOPs for right and left eye were 36 mm Hg and 45 mm Hg, respectively. Gonioscopy indicated neovascularization of the angle and iris in both eyes (Fig. 2A). Funduscopic examination revealed a preretinal hemorrhage on the left eye and neovascularization of both optic discs. MTMT was performed for the neovascular glaucoma of both eyes and intravitreal anti-VEGF injection was administered to the left eye. One day later, visual acuity of the left eye had decreased to hand motion because of severe hyphema, and IOP was 44 mm Hg (Fig. 2B). AGV implantation and intracameral air injection was planned. Following tube insertion, air was injected into the anterior chamber with an 80% to 90% air fill though a paracentesis site. At the end of surgery, the tube tip was positioned in an air bubble. At one day postoperatively, left eye visual acuity was 0.08 and IOP was 21 mm Hg. Slit lamp examination revealed the tube opening was in air, far from blood clots; it appeared the air had pushed blood clots to the peripheral angle of the anterior chamber (Fig. 2C). Three days postoperatively, air bubbles were much reduced and hyphema was diminished (Fig. 2D). During the 6 months of follow-up, left eye IOP remained stable on one topical medication (Tables 1 and 2).

### 2.3 Case 3

**Figure 3:**
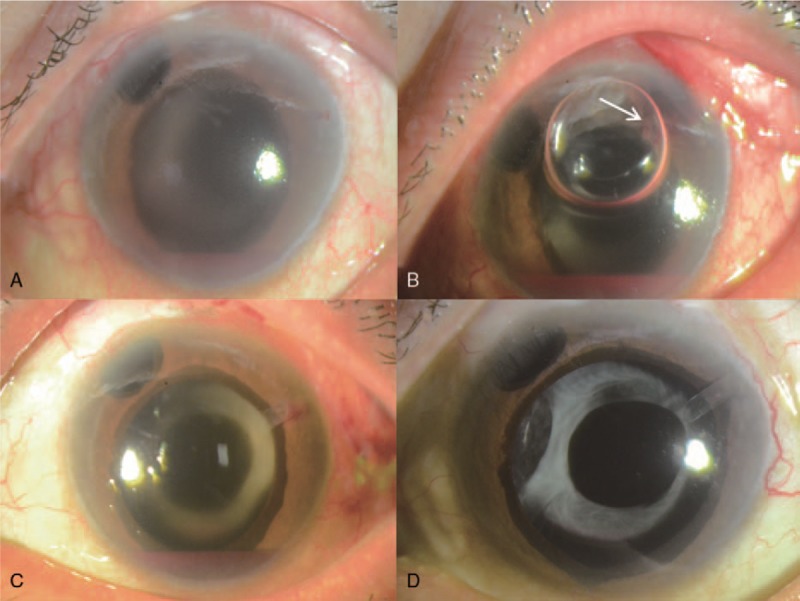
Case 3: 53/M (A) neovascular glaucoma with hyphema and vitreous haemorrhage. (B) Tube opening where air contacted was patent (white arrow) although other part of tube was covered with blood clots on the first day after AGV implantation and intracameral air injection. (C and D) Blood clots around the tube and hyphema decreased gradually at 9 days and disappeared on 2 months after the surgery. AGV = Ahmed glaucoma valve.

A 53-year-old man had been treated for neovascular glaucoma of the left eye and proliferative diabetic retinopathy of both eyes for 3 years, and had undergone pan-retinal photocoagulation and trabeculectomy of the left eye for neovascular glaucoma. His BCVA was 0.9 for the right eye and light perception for the left. Intraocular pressures of right and left eyes were 14 mm Hg and 40 mm Hg, respectively. Extensive neovascularization of iris, ectropion uvea, hyphema, and vitreous hemorrhage were present in the left eye (Fig. 3A). MTMT and intravitreal anti-VEGF injection with paracentesis were performed, but AGV implantation was subsequently planned because IOP was not controlled. During the surgery, intracameral air was administered with an 80% to 90% air fill because bleeding from the angle encroached the tube. In addition, hyphema and vitreous hemorrhage were still observed. On the first postoperative day, hyphema and pretubal blood clots were observed, but despite a decrease in the size and mobility of the air bubble, the tube remained open (Fig. 3-B). IOP was 10 mm Hg on the first postoperative day. Hyphema and vitreous hemorrhage disappeared over 2 months and visual acuity increased to 0.15 (Fig. 3C and D). IOP has been stable on 2 topical medications for the 6 months since the surgery (Tables 1 and 2).

### 2.4 Case 4

**Figure 4:**
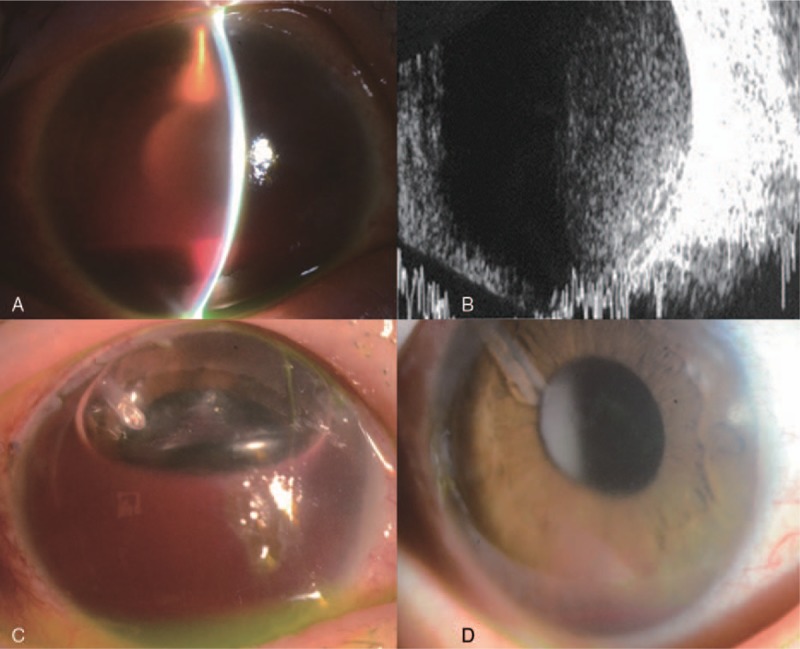
Case 4: 35/F, (A) neovascular glaucoma with severe hyphema. (B) Vitreous hemorrhage on preoperative B-scan. (C) The tube was in air with stable IOP on the first postoperative day after AGV implantation and intracameral air injection. (D) Hyphema was decreased with the height of less than 1 mm but IOP at one month after the surgery. AGV = Ahmed glaucoma valve, IOP = intraocular pressure.

A 35-year old female was referred for severe right eye pain due to recurrent IOP elevation. She had pars plana vitrectomy and cataract surgery for proliferative diabetic retinopathy and vitreous hemorrhage and cataract 4 months ago. Hyphema and vitreous hemorrhage were evident (Fig. 4A and B), and right eye visual acuity was hand motion with an IOP of 46 mm Hg. MTMT and anti-VEGF injection with paracentesis could not decrease IOP. Severe ocular pain was repeated. Thus, AGV implantation and intracameral air injection was performed simultaneously. The anterior chamber was filled with an 80% to 90% air fill. On the first postoperative day, IOP was 13 mm Hg and an air-fluid level was noted in the anterior chamber. The tube opening was in air and free of tube obstruction (Fig. 4C). One month after surgery, right eye visual acuity improved to 0.08 with decreased hyphema and IOP was 15 mm Hg without any IOP lowering medications (Fig. 4D, Tables 1 and 2).

## 3 Discussion

This study has shown that intracameral air injection may be a simple method that has a preventive effect on tube obstruction by blood clots in high risk cases including neovascular glaucoma with intraocular hemorrhages. The effect of intracameral air injection is primarily based on the physical property of air which means that air does not mix with a fluid. We filled about 50% of the anterior chamber in the first case and about 80% to 90% in the other cases. However, the preventive effect of air could be observed in all cases. We hypothesized it as follows. When the tube opening is in air, as was observed in cases 2 and 4, a large air inhibited blood from going near the tube opening, and thus, air could reduce the risk of blood clot formation in the tube. On the other hand, in cases 1 and 3, tube openings were only intact although blood clots were formed around the tubes. From this observation, we supposed that as the size of the air decreased, the air bubble could move and contact the tube opening. This intermittent contact between the air bubble and tube opening might interfere with blood clot formation.

The introduction of anti-VEGF decreased the risk of postoperative bleeding in patients with neovascular glaucoma.^[11]^ Even though an anti-VEGF injection is administered before glaucoma drainage implantation surgery, tube blockage rate was not significantly decreased according to a meta-analysis.^[11]^ It may be because the complication would be associated with a number of factors including the severity of neovascularization of the iris and the anterior chamber angle, the severity of underlying diseases, the level of baseline IOP, and changes in perioperative IOP.^[13]^ Moreover, the risk of tube obstruction with blood clots cannot be completely predicted, especially in severe neovascular glaucoma cases with intraocular hemorrhage. Therefore, effective methods to prevent the tube obstruction with blood clots are still needed.

Management options for blocked tube include conservative management with topical and oral steroid, T-PA, flushing the tube and, and Nd-YAG laser membranectomy. Conservative measures sometimes fail to lyse clots. Conventional surgical procedures to remove a blood and flush the tube need a return to the operating room and may precipitate new bleeding or fibrin formation. Significant complications including severe hyphema, hypotony, and anterior chamber flattening was reported to occur about 10% after T-PA administration.^[12]^ Nd YAG laser membranectomy in blocked tubes after glaucoma tube shunt surgery was also reported to be related with a relatively high rate of subsequent reblockage.^[1]^ In this regard, intracameral air injection needs to be considered as a feasible option to reduce the risk of tube obstruction with blood clots in high risk cases.

Limitations of this study should be mentioned. First of all, the number of cases was small and its design was noncomparative, and thus, we cannot rule out the possibility that tube obstruction would not have been developed without an intracameral air injection. Nevertheless, we believe intracameral air injection more certainly lowers the risk of tube obstruction by blood clots due to its physical property. Second, the air-induced pupillary block has been previously described,^[14–17]^ and it would occur when complete air fill of the anterior chamber presses the iris against the lens and blocks flow through the pupil.^[18]^ The amount of air increased from 50% in the first case to 80–90% in anterior chamber in the other cases because of the severity of intraocular hemorrhage. However, it would be careful not to fill air into the anterior chamber completely for the prevention of tube obstruction by blood clots. In addition, the patients should be instructed to avoid prone position, which could cause pupillary block by air migrating back to the pupil after surgery. Finally, this procedure does not alter the long term outcome after glaucoma drainage implant surgery in neovascular glaucoma. Nevertheless, it may be clinically relevant to have a patent tube in early postoperative period for the management of neovascular glaucoma. The intact flow of the tube in early postoperative period could reduce blood clot formation around the tube by evacuating it gradually when recurrent hemorrhage would occur. However, further large-scaled studies should be followed to determine the long term effect of the procedure. In conclusion, we report 4 cases which suggested that intracameral air injection may be a simple and effective means of preventing tube obstruction of AGV implant by blood clots in high risk cases such as neovascular glaucoma with severe intraocular hemorrhage. To the best of our knowledge, this is the first report to suggest the possibility that intracameral air may help minimize tube obstruction of AGV implant with blood clots in neovascular glaucoma. Further studies are warranted to confirm the effect of this feasible procedure.

## Acknowledgment

None of the authors have any commercial or proprietary interests in any of the instruments or materials used in this work.
